# Recent progress in toxicogenomics research in South Korea

**DOI:** 10.1186/1753-6561-3-s2-s6

**Published:** 2009-03-10

**Authors:** Tae-Hoon Chung, Jin-Ho Yoo, Jae-Chun Ryu, Yang-Seok Kim

**Affiliations:** 1 Korea Biobank, Center for Genome Science, Korea National Institute of Health, Korea Centers for Disease Control and Prevention, 194 Tongil-lo, Eunpyung-gu, Seoul, 122-701, Republic of Korea; 2 Cancer Metastasis Research Center, Yonsei University College of Medicine, 260 Seongsanno, Seodaemun-gu, Seoul, 120-752, Republic of Korea; 3 Brain Korea 21 Project for Medical Science, Yonsei University College of Medicine, 260 Seongsanno, Seodaemun-gu, Seoul, 120-752, Republic of Korea; 4 Toxicology Laboratory, Korea Institute of Science and Technology, PO Box 131, Cheongryang, Seoul, 130-650, Republic of Korea; 5 College of Oriental Medicine, Kyung Hee University, Hoegi-dong, Dongdaemun-gu, Seoul, 130-701, Republic of Korea

## Abstract

**Background:**

The importance of toxicogenomics was recognized early in Korea and a group of researchers was trying to build up a research infrastructure and educational system. However, since the scale of the Korean pharmaceutical industry, which was expected to play the key role in toxicogenomics was small compared to that of advanced countries, industry-sponsored large-scale research projects and supporting infrastructures have been lacking in Korea.

**Results:**

To improve this situation, the Korean government has exerted special efforts to promote toxicogenomics research and development the last few years as an initiative to stimulate a premature drug development industry on par with global competition and launched several large scale research projects recently. Researchers are also trying to keep pace with government efforts by organizing local scientist groups, training young toxicogenomics scientists, and widening the toxicogenomic research efforts to environmental toxicity as well. Research and development from bioinformatics and genomics venture companies are also contributing to uplifting the competitiveness of the toxicogenomics industry.

**Conclusion:**

Toxicogenomics in Korea is making steady progress in many directions. It is gaining ground by government and related industries as well, the research is diversified to embrace environmental genomics, and local research groups are making strategic links to international research groups such as the MicroArray Quality Control (MAQC) consortium. We expect the advancement of the Korean toxicogenomics research program will be beneficial not only to the local society alone, but also to international scientists as a whole.

## Background

The importance of toxicogenomics techniques in the accurate prediction of the toxicity of chemicals under pre-clinical evaluation is well-appreciated already.[1] In particular, systems toxicology, which takes advantage of the systems biological approaches, is presenting the possibility of rapid and accurate toxicity prediction by integrating various "omics"-based techniques such as genomics, transcriptomics, proteomics, metabolomics etc.[2] However, to take the full benefits of systems toxicology, there are still issues to be resolved such as the standardization and accumulation of different omics data and the promotion of open access to various distributed data archives.[3] Of course, the resolution of these issues demands concentrated efforts among researchers around the world. In Korea as well, research efforts are expended from various sides including government, academic organizations, and industry, realizing the importance of toxicogenomics and its down-stream effects. In this paper, we will briefly review efforts to promote toxicogenomics in Korea by focusing on the three sides iteratively.

## Results

### Government sector

**Figure 1:**
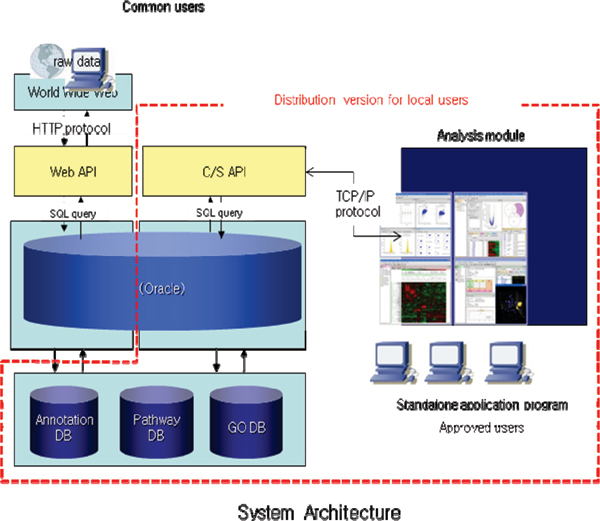
Architecture of KOTIS whole system

**Figure 2:**
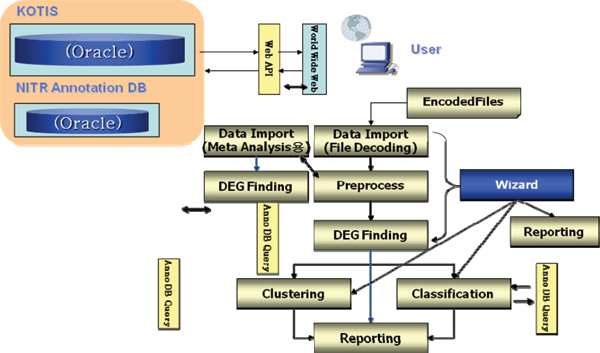
Whole data flow chart

The National Institute of Toxicological Research (NITR) [4] is a leading government research agency in the field of toxicogenomics in Korea. With a long-term mission to develop toxicogenomics-based toxicity and safety assessment techniques, NITR is conducting various research and development projects. Among them is the construction of s toxicoinformatics infrastructure called KOTIS, an acronym of Korea Toxicoinformatics Integrated System, which is carried out jointly with ISTECH Inc.[5]. KOTIS which is modeled against ArrayTrack [6] and CEBS [7] is composed of a database system and its analysis programs. It will archive all government-funded toxicogenomics research results in Korea and disseminate them back to interested researchers (Figure 1). In particular, not only does it stores and redistributes the basic experimental data but it also allows users to download selected data needed for meta-analysis after re-processing archived expression data on top of quality assessment results. It will be able to deal with most of the expression platforms currently used by toxicogenomics researchers. Analysis programs are designed to seamlessly work with the KOTIS database under the client/server environment. The actual implementation focuses not only on conventional techniques for expression data analysis such as quality assessment, significant gene finding, clustering and classification, but also on meta-analysis – a recent hot research issue in toxicogenomics. For meta-analysis, we will allow more choices to users by implementing parametric and non-parametric statistical techniques as well as the simple "gene list agreement" method. This will allow users to flexibly select an analysis method that is appropriate for the characteristics of the data (Figure 2). KOTIS is expected to level up the toxicogenomics research and development in Korea tremendously. Besides the construction of toxicoinformatic infrastructure, NITR is also conducting various transcriptomics projects to accumulate data on toxicity-induced expression changes in response to chemicals in model organisms by collaborating with academic researchers. NITR has already accumulated expression profiles against ~100 toxic materials through 22 independent projects in the year 2007. In addition, NITR is also making great efforts to establish international research cooperations with global research organizations like the National Center for Toxicological Research (NCTR).

The Korea Institute of Toxicology (KIT) [8] is another government agency that is operating a toxicogenomics special team since year 2002. The toxicogenomics team is performing researches on 5 specialized areas (genomic DNA (SNP), RNA & gene expression, proteomics, cell culture (siRNA), and histopathology & blood biochemistry) and is specifically focusing on hepatotoxicity, nephrotoxicity, and reproductive toxicology. Furthermore, by conducting toxicogenomics research on the possibility of using monkey's peripheral blood mononuclear cells (PBMC) as a surrogate tissue, it is expected to provide novel research results on toxicogenomics which can be quite complementary to conventional ones that have been obtained mostly in mice and rats.

Besides, the Korean Ministry of Environment recently launched another large scale project aim at the utilization of toxicogenomics techniques in evaluating environmental toxicity. It is expected that government-initiated toxicogenomics research drives will be expanded progressively.

### Academic sector

Led by academic researchers, the establishment of "The Korean Society of Toxicogenomics and Toxicoproteomics"(KSTT) [9] in the year 2004 became the cornerstone event for toxicogenomics in Korea. Its official journal "Molecular and Cellular Toxicology"(MCT) has been the central communication channel among local toxicogenomics researchers since its inception and became a Science Citation Index Expanded (SCIE) journal in just two years. KSTT is making its best efforts to improve the contents of MCT by inviting leading toxicogenomics scientists to the editorial board and imposing stringent acceptance criteria [10-12]. KSTT also places special emphasis on opening global academic connections and addressing local research results world wide by hosting international workshops annually. Expanding the human resource basis for toxicogenomics is an important mission of KSTT and it is trying to educate the importance, and new developments of toxicogenomics to young researchers and graduate-level students through numerous workshops and topical schools. KSTT already gained 500+ regular members in the year 2006. All these concerted efforts to enhance public awareness for toxicogenomics eventually culminated in launching the large scale project called "Establishment and Application of Toxicogenomics for Assessment and Prediction of Toxicity in Environmental Aspects" by the Ministry of Environment. We expect the proactive role played by KSTT will definitely be beneficial in forming a solid foundation for toxicogenomics research and development by expanding a professional human resource basis, promoting public awareness and support, and motivating communcations between domestic and international researchers from the long-term point of view.

### Business sector

One particular aspect of the pharmaceutical industry in Korea is that the scale of pharmaceutical companies is so small that investment for a drug development infrastructure and related technology is almost negligible compared to that of economically-developed countries. Therefore, it is unimaginable to expect investments in toxicogenomics research from local pharmaceutical industry. However, this does not mean that there is no toxicogenomics research activity from the business sector. Ironically, the lack of investments in toxicogenomics from established companies became a good opportunity for small genomics and bioinformatics venture companies. They develop toxicogenomics technologies and products such as toxicity diagnosis chips through strategic alliances. One representive example is the three-tiered alliance by Genocheck [13] a genomics-oriented company, ISTECH a bioinformatics company, and Shinwon Science [14] a pre-clinical contract research organization (CRO). The alliance succeeded in developing the hepatotoxicity diagnosis chip through the analysis of expression patterns of hepatotoxicity molecules and the three companies are continuing toxicogenomics research by applying information and techniques acquired during the cooperation. Especially, ISTECH is playing an important role in building up the national toxicoinformatics infrastructure and developing meta-analysis techniques through a joint research effort with NITR.

## Conclusion

Korean toxicogenomics is still in its infancy compared to the US, EU or other advanced countries. However, government-driven and well-designed investments are expected to make critical contributions to toxicogenomics research and development. Also, the establishment of KSTT and its drive to research communication and education by academic scientists, and the continued research investment and product development by venture companies are also expected to promote domestic toxicogenomics research and development.

## Competing interests

The authors declare that they have no competing interests.

## Authors' contributions

THC and YSK collected basic information and prepared the manuscript. JHY and JCR provided information for the manuscript.
